# Ferromagnetic mechanism in organic photovoltaic cells with closed-shell structures

**DOI:** 10.1038/s41598-017-09004-8

**Published:** 2017-08-21

**Authors:** Liu Yang, Shixuan Han, Xiaolei Ma, Wei Qin, Shijie Xie

**Affiliations:** 1 School of Physics, State Key Laboratory of Crystal Materials, Shandong University, Jinan, China; 2 College of Physics and Engineering, Qufu Normal University, Qufu, China

## Abstract

We construct a model to reveal the spin polarization or ferromagnetism observed in organic composite nw-P3HT/C_60_ with closed-shell structures. Different from the organic ferromagnets with open-shell structures, the ferromagnetism of nw-P3HT/C_60_ comes from the charge transfers from the polymer to the small molecules. The transferred electrons become spin polarized and they are coupled together through the holes in the polymer. Finally, a ferromagnetic order appears in the pure organic composite. The magnetic moment of the system is mainly provided by the spin polarized small molecules. The magnetization is dependent upon the density of the transferred charges, which is consistent to our experimental observations. Our investigation also shows that some new spin phenomena may appear in excited states for organic semiconductors which is absent in the ground states.

## Introduction

Organic photovoltaic (OPV) cells are emerging as a clean and sustainable source of energy and are expected to play a major role in meeting the global energy challenge^1, 2^. The general active layer structure of an organic photovoltaic device is donor-acceptor (D-A) heterojunction^3^. In organic materials, the primary photo-excited species are excitons, i.e., bound electron-hole pairs^4^. Photo-induced charge transfers in D-A heterojunctions provide a high power conversion efficiency^5, 6^. To improve the efficiency of the OPV cells, many efforts were made. For example, doping the active layer with spin 1/2 radicals^7^ and inserting an ultrathin film of a ferroelectric co-polymer^8^. The influences of the singlet and triplet excitons on the OPV cells were also investigated^9^. Recently, in the experiments Li *et al*. used an organic open-shell molecule in which there is only one electron in the highest occupied molecular orbital (HOMO)^10, 11^. Organic ferromagnets are also a kind of open-shell magnetic molecules, which show ferromagnetic ground state because of the unpaired electron in the side radical^12, 13^. However, open-shell molecules with neutral radicals are quite unstable generally, while closed-shell molecules are more widely used in reality. For a closed-shell molecule, there are two electrons in the HOMO. Can we realize magnetism in a closed-shell molecule, and how to achieve it?

In 2012, Ren *et al*. investigated the magnetic properties of organic charge-transfer heterojunction. They found the excitonic room-temperature ferromagnetism in semiconducting single crystal poly-3(hexylthiophene) nanowires (nw-P3HT) doped with fullerene (C_60_)^14^, which are wildly used in OPV cells^15, 16^. It is known that the pristine nw-P3HT and C_60_ single component are of closed-shell structure, and both of them do not exhibit any ferromagnetic behavior. The appearance of ferromagnetism in nw-P3HT/C_60_ should come from the charge transfer between nw-P3HT and C_60_. Especially it is revealed that the saturated magnetization has an apparent enhancement once under light illumination. For example, the magnetization in darkness is about 12 emu/cm^3^. Illumination of this sample with a 615 nm laser makes the moment increase to 30 emu/cm^3^. Later in 2014, the excited ferromagnetism in a nano-carbon bulk heterojunction device consisting of C_60_ and semiconducting single-walled carbon nanotubes was also demonstrated^17^. These reports tell us that some new magnetic phenomena may appear in organic charge transfer composites in the excited state that may be absent in the ground state. Further, it was found that the magnetization changes under manipulation of electric field and mechanical stress, so it seems that the organic charge transfer composites have the characteristics of multiferroics^18^. At the same time, the similar phenomena were also found in nw-P3HT/PCBM charge transfer composite^19^. All these experimental results imply that organic charge transfer composites have more abundant properties than their single components.

For either nw-P3HT or C_60_, they have a closed-shell structure. To present spin signal, we have to break the closed-shell structure through charge doping or photo-exciting. Recently, we investigated the charge transfer states (CTSs) or excited states (EXs) and their spin polarization in organic charge transfer composites such as nw-P3HT/C_60_ with a well-known tight binding model^20^. By including the electron-electron (e-e) interaction and spin-orbit coupling (SOC), it was found that the CTSs or EXs are spin mixed and polarized which is different from the pure singlet or triplet ones. For example, due to the different electron-phonon (e-ph) coupling of the acceptor from the donor, the electron and hole in acceptor and donor respectively will have different spin polarization intensities, which result in a net spin for an inter-molecule exciton. These spin polarized CTSs or EXs provide the basic species for the organic ferromagnetism. Then we suggested a possible mechanism for the origin of excited ferromagnetism. These CTSs or EXs coupled through nw-P3HT, which makes the spins on C_60_ arrange parallel. This model is similar to that of organic ferromagnets, such as poly-BIPO, which explain the ferromagnetic ground state of the open-shell magnetic molecule qualitatively. Up to now, it is still not clear how these magnetic moments organized together explicitly.

In this paper, we will study the ferromagnetism of organic molecules with closed-shell structures. Referring to the experiments work on nw-P3HT/C_60_^14^ and nw-P3HT/PCBM^19^, we construct a composite consisting of a polymer and small molecules.

## Model

**Figure 1:**
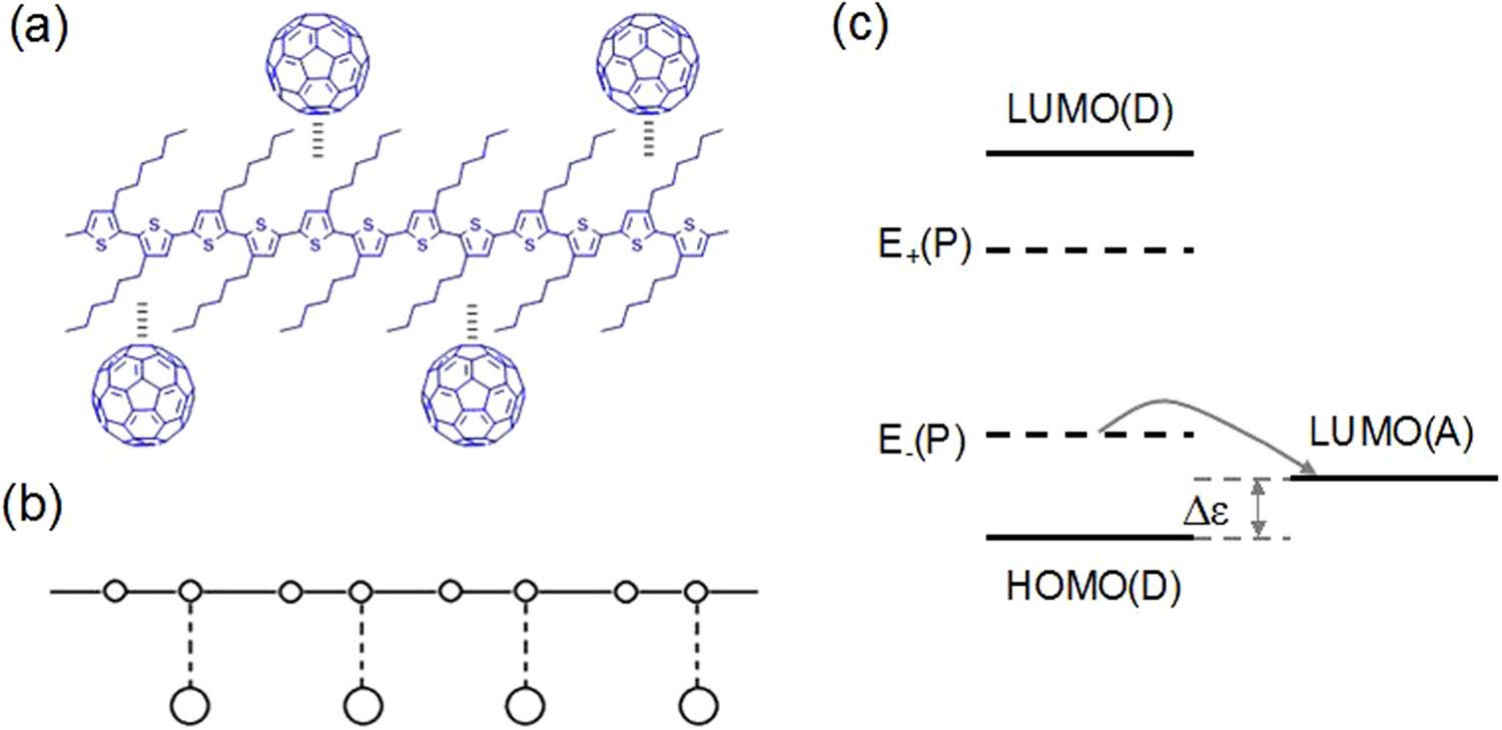
Schematic diagrams of (**a**) nw-P3HT/C_60_ composite, (**b**) the model and (**c**) the energy levels (the dashed lines are the polaron energy levels).

In an OPV device, a D-A heterojunction is synthesized. The donors are conjugated polymers, and the acceptors are small molecules. A schematic figure is shown in Fig. 1a. We model the composite with tight-binding approximation as showed in Fig. 1b. The polymer molecule is described with extended Su-Schrieffer-Heeger (SSH) model^21, 22^. The small molecule is considered as a radical attached on the polymer through the weak Van der Waals force. In the ground states, both the polymer and the small molecules are closed-shell, which means that neither of them have net spin polarization. But if there exists charge transfer between them, the closed-shell structures will be broken.

The Hamiltonian is written as

1 $$H={H}_{D}+{H}_{A}+{H}_{DA}$$

where the first term is for the polymer, the second for the small molecules and the last for their coupling. In the tight-binding approach, they are given respectively by,

2 $$\begin{array}{rcl}{H}_{D} & = & -\sum _{i,s}{E}_{D}{d}_{i,s}^{+}{d}_{i,s}-\sum _{i,s}{t}_{i,i+1}({d}_{i,s}^{+}{d}_{i+1,s}+{d}_{i+1,s}^{+}{d}_{i,s})\\  &  & +\sum _{i}{U}_{D}{d}_{i,\uparrow }^{+}{d}_{i,\uparrow }{d}_{i,\downarrow }^{+}{d}_{i,\downarrow }-\sum _{i}{t}_{so}({d}_{i+1,\uparrow }^{+}{d}_{i,\downarrow }+{d}_{i,\downarrow }^{+}{d}_{i+1,\uparrow }-{d}_{i+1,\downarrow }^{+}{d}_{i,\uparrow }-{d}_{i,\uparrow }^{+}{d}_{i+1,\downarrow })\\  &  & +\,\frac{1}{2}K\sum _{i}{({u}_{i+1}-{u}_{i})}^{2}\end{array}$$

3 $${H}_{A}=-\,\sum _{j,s}{E}_{A}{a}_{j,s}^{+}{a}_{j,s}+\sum _{i}{U}_{A}{a}_{j,\uparrow }^{+}{a}_{j,\uparrow }{a}_{j,\downarrow }^{+}{a}_{j,\downarrow }$$

4 $${H}_{DA}=-\,\sum _{ < i,j > ,s}{t}_{DA}({d}_{i,s}^{+}{a}_{j,s}+{a}_{j,s}^{+}{d}_{i,s})-\sum _{ < i,j > }J{\overrightarrow{s}}_{i}\cdot {\overrightarrow{s}}_{j}$$

where *E*_*D*(*A*)_ is the on-site energy of the *π*-electrons on the polymer (small molecules), which determine the energy offset between donor and acceptor. ${d}_{i,s}^{+}$(${d}_{i,s}$) and ${a}_{i,s}^{+}$ (${a}_{i,s}$) is the electron creation (annihalation) operator at site *i* with spin *s* at the polymer and the small molecules, respectively. ${t}_{i,i+1}={t}_{0}-\alpha ({u}_{i+1}-{u}_{i})-{(-1)}^{i}{t}_{e}$ is the transfer integral of *π* electrons between sites *i* and *i* + 1 along the polymer chain. *t*_0_ is the electron transfer integral in a uniform lattice. *α* is the e-ph coupling coefficient between neighboring sites due to the site displacement. *u*_*i*_ is the deviation of site *i* from the uniform configuration. *t*_*e*_ is the Brazovskii-Kirova symmetry breaking term, which indicates the non-degeneracy of polymer^23^. *U*_*D*(*A*)_ is the Hubbard *e-e* interaction^24^. The fourth term in Eq. (2) is the SOC^25, 26^, and the last term denotes the elastic energy due to the lattice displacement. *K* denotes the lattice elastic constant. *t*_*DA*_ means the intermolecular electron transfer integral between the polymer and the corresponding small molecule. As the small molecules interacting with the polymer through the weak Van der Waals force, *t*_*DA*_ is much smaller than the intramolecular integral *t*_0_. The last term in Eq. (4) shows the Heisenberg spin-spin coupling^27^ between the polymer and the small molecules with intensity *J*. $\sum _{ < i,j > }$ denotes the summation of the corresponding sites in the polymer and the small molecules. The *π*-electron spin is defined with creation and annihilation operator as ${\vec{s}}_{i}=\sum _{\sigma ,\sigma ^{\prime} }{c}_{i,\sigma }^{+}({\vec{\sigma }}_{\sigma \sigma ^{\prime} }/2){c}_{i,\sigma ^{\prime} }$ ^28^, where ${\vec{\sigma }}_{\sigma \sigma ^{\prime} }$ is the Pauli matrix^29^. If the polymer and the small molecules are in closed-shell, we have ${\vec{s}}_{i}=0$ in the pristine ground state, and there is no spin coupling. If the closed-shell structures are broken due to charge transfer, spin polarization may appear and there will be an apparent spin dependent coupling, which is much important for the magnetism of the whole system.

For simplicity, we treat the *e-e* interaction and the spin-spin coupling with mean field approximation as,

5 $${d}_{i\uparrow }^{+}{d}_{i\uparrow }{d}_{i\downarrow }^{+}{d}_{i\downarrow }\approx [\langle {d}_{i\uparrow }^{+}{d}_{i\uparrow }\rangle {d}_{i\downarrow }^{+}{d}_{i\downarrow }+\langle {d}_{i\downarrow }^{+}{d}_{i\downarrow }\rangle {d}_{i\uparrow }^{+}{d}_{i\uparrow }-\langle {d}_{i\uparrow }^{+}{d}_{i\uparrow }\rangle \langle {d}_{i\downarrow }^{+}{d}_{i\downarrow }\rangle ]-[\langle {d}_{i\uparrow }^{+}{d}_{i\downarrow }\rangle {d}_{i\downarrow }^{+}{d}_{i\uparrow }+\langle {d}_{i\downarrow }^{+}{d}_{i\uparrow }\rangle {d}_{i\uparrow }^{+}{d}_{i\downarrow }-\langle {d}_{i\downarrow }^{+}{d}_{i\uparrow }\rangle \langle {d}_{i\uparrow }^{+}{d}_{i\downarrow }\rangle ]$$

6 $${\vec{s}}_{i}\cdot {\vec{s}}_{j}\approx \langle {\vec{s}}_{i}\rangle \cdot {\vec{s}}_{j}+\langle {\vec{s}}_{j}\rangle \cdot {\vec{s}}_{i}-\langle {\vec{s}}_{i}\rangle \cdot \langle {\vec{s}}_{j}\rangle $$

Where we employ the Wick theorem^30^, and the spin exchange coupling is involved in the second term of Eq. (5). In this case, each state *ψ*_*μ*_ is spin mixing and is expressed in the basis of Wannier wave function as^20, 31^,

7 $${\psi }_{\mu }=(\begin{array}{c}\sum _{i}{Z}_{\mu ,i,\uparrow }|i\rangle \\ \sum _{i}{Z}_{\mu ,i,\downarrow }|i\rangle \end{array})$$

where *Z*_*μ*,*i*,*s*_ means the probability amplitude of state *ψ*_*μ*_ with spin *s* at site *i*. Together with the eigen-energy *ε*_*μ*_, it is determined by following eigen-equations,

8 $$\{\begin{array}{rcl}{\varepsilon }_{\mu }^{D}{Z}_{\mu ,i,s} & = & ({E}_{D}+{U}_{D}{n}_{i-s,i-s}){Z}_{\mu ,i,s}-{t}_{i-1,i}{Z}_{\mu ,i-1,s}-{t}_{i,i+1}{Z}_{\mu ,i+1,s}-{U}_{D}{n}_{is,i-s}{Z}_{\mu ,i,-s}\\  &  & \mp \,\,{t}_{so}({Z}_{\mu ,i-1,-s}-{Z}_{\mu ,i+1,-s})-{t}_{DA}{Z}_{\mu ,j,s}-\frac{J}{4}({n}_{js,js}-{n}_{j-s,j-s}){Z}_{\mu ,i,s}\\ {\varepsilon }_{\mu }^{A}{Z}_{\mu ,j,s} & = & ({E}_{A}+{U}_{A}{n}_{j-s,j-s}){Z}_{\mu ,j,s}-{U}_{A}{n}_{js,j-s}{Z}_{\mu ,j,-s}-{t}_{DA}{Z}_{\mu ,i,s}-\frac{J}{4}({n}_{is,is}-{n}_{i-s,i-s}){Z}_{\mu ,j,s}\end{array}$$

where ${n}_{is,js\text{'}}=\sum _{\mu }^{\prime} {Z}_{\mu ,i,s}^{\ast }{Z}_{\mu ,j,s\text{'}}$ is the spin-charge density matrix. $\sum _{\mu }^{\prime}$ means sum over all the occupied states. $\mp$ takes ‘−’ for *s* = ↑ and ‘+’ for *s* = ↓. The lattice configuration in Eq. (8) is determined by the static equilibrium condition through minimizing the total energy,

9 $${u}_{i+1}-{u}_{i}=\frac{2\alpha }{NK}\sum _{\mu ,i,s}^{\prime} {Z}_{\mu ,i,s}{Z}_{\mu ,i+1,s}-\frac{2\alpha }{K}\sum _{\mu ,s}^{\prime} {Z}_{\mu ,i,s}{Z}_{\mu ,i+1,s}$$

By combining Eqs (8) and (9), electronic states and lattice configurations are solved self consistently. Firstly, eigen-value Eq. (8) is solved with an initial configuration {*u*_*i*_}, and then substitute the eigen-states into Eq. (9) to find a new configuration. The calculations will be repeated with this new configuration. The criterion for the self-consistency is that the difference between values of {*u*_*i*_} from two successive iterations is less than 10^−7^ Å.

To check the stability and the properties of the system, we calculate the total energy *E*_*t*_ of the system and the spin distribution. The total energy consists of the electron energy *E*_*e*_ as well as lattice energy *E*_*l*_ given by,

10 $$\{\begin{array}{c}{E}_{e}=\sum _{\mu }^{\prime} {\varepsilon }_{\mu }-\sum _{i}{U}_{D}({n}_{i\uparrow ,i\uparrow }{n}_{i\downarrow ,i\downarrow }-{n}_{i\downarrow ,i\uparrow }{n}_{i\uparrow ,i\downarrow })-\sum _{j}{U}_{A}({n}_{j\uparrow ,j\uparrow }{n}_{j\downarrow ,j\downarrow }-{n}_{j\downarrow ,j\uparrow }{n}_{j\uparrow ,j\downarrow })+\frac{1}{4}\sum _{ < i,j > }J{m}_{i}{m}_{j}\\ {E}_{l}=\frac{1}{2}\sum _{i}K{({u}_{i+1}-{u}_{i})}^{2}\\ {E}_{t}={E}_{e}+{E}_{l}\end{array}$$

while the spin distribution is ${m}_{i}={n}_{i\uparrow ,i\uparrow }-{n}_{i\downarrow ,i\downarrow }$ (in unit of *ћ*/2).

Numerical calculation is carried out in a system with a polymer chain containing 100 CH units. The small molecules are attached to the polymer unit alternatively. If we do not consider the boundary effect, the final result should be independent of the polymer length. Also we can design different adsorption of the small molecules to the polymer. The model parameters are *t*_0_ = 2.5 *eV*, *α* = 4.1 *eV*/Å, *t*_*e*_ = 0.05 *eV*, *K* = 21 *eV*/Å^2^, which are usually chosen for polyacetylene^32^. *U*_*D*_ = *U*_*A*_ = 1.0 *eV*, *t*_*s*o_ = 0.01 *eV*. Considering that the coupling between the polymer and the small molecules is weak, we choose *t*_*DA*_ = 0.1 *eV* ^33^ and *J* = −0.1 *eV*. We assume the antiferromagnetic coupling between spins in the polymer and the small molecules, which does not affect our physical picture and conclusions. Some parameters are changed to discuss their effects on the properties of the system.

## Results and Discussion

**Figure 2:**
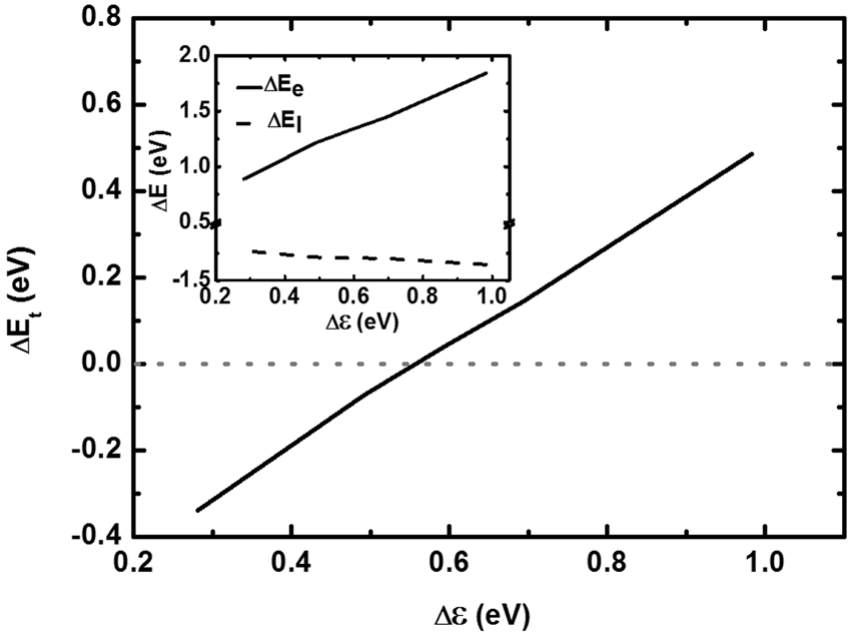
Dependence of Δ*E*_*t*_ on Δ*ε*. Insert: dependence of Δ*E*_*e*_ and Δ*E*_*l*_ on Δ*ε*, respectively.

In the pristine state, both the polymer and the small molecules are in closed-shell. When they are combined together, charge transfer may take place, which depends upon the relative position of the HOMO and the lowest unoccupied molecular orbital (LUMO) levels. As shown in Fig. 1c, the HOMO level of the polymer is lower than the LUMO of the small molecule, there should be no charge transfer. However, it is found that the actual situation is not this. The polymer is soft material, which contains charged polarons. If the polaron energy levels are higher than the LUMO levels of the small molecules, the charge will transfer from the polaron level to the small molecule LUMO level. There are two cases for the formation of polarons in the polymer: one is unintentionally doping or thermal injection from environment^34^, and the other one is the excited tunneling of electrons from the polymer to the small molecules. To show the spontaneous charge transfers, we calculate the electron energy, the lattice energy and the total energy of the system after and before photoexcitation, respectively. These quantities are defined as Δ*E*_*j*_ = *E*_*j*_(CT) − *E*_*j*_(0) with *j* = *e*, *l*, *t*. *E*_*j*_(CT) and *E*_*j*_(0) mean energy after and before photoexcitation respectively. Taking *E*_*A*_ − *E*_*D*_ = 0.1 *eV*, it is obtained Δ*E*_*e*_ = 1.18 *eV* and Δ*E*_*l*_ = −1.38 *eV*. Δ*E*_*e*_ < 0 means that the electronic energy will increase after charge transfer, and Δ*E*_*l*_ < 0 means that the lattice energy will decrease after charge transfer. While Δ*E*_*t*_ = −0.2*e* < 0 means that the system is energy favorable for charge transfer state. Therefore, for organic composites, charge transfer may take place even the HOMO level of the polymer is lower than the LUMO of the small molecules, which is difference from inorganic semiconductors with rigid band structures. Defining Δ*ε* is the difference between the HOMO level of the polymer and LUMO one of the small molecules. When an electron transfers from the polymer to the small molecules, it needs to absorb energy Δ*ε*, so the electron energy increases. But due to the strong e-ph coupling, the charged polymer will release its lattice energy to form a localized state (polaron). Δ*E*_*t*_ dependent Δ*ε* is shown in Fig. 2, where we see that charge transfer may take place once Δ*ε* < 0.55 *eV*.

**Figure 3:**
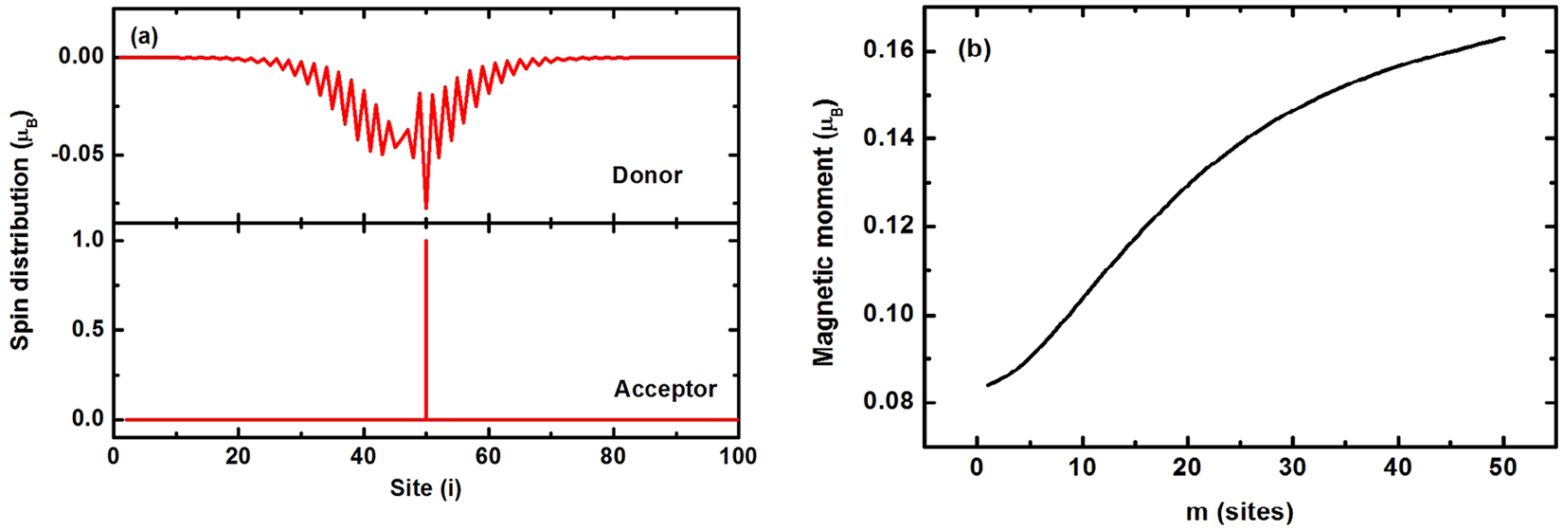
**(a)** Spin distribution of the system when one electron transfers from the polymer to the small molecules. **(b)** Dependence of the magnetic moment on electron distribution in the small molecules.

Under light illumination, substantial electrons transfer from the polymer to the small molecules. In this case, the closed-shell structures of either polymer or the small molecules are broken. Then we consider the spin polarization of system. When one electron transfers from the polymer to the small molecules, it is obtained that the system is most stable when the transferred electron is localized in one small molecule, while the left hole in the polymer forms a polaron which is spin spontaneous polarized^31^. Due to the *e-e* interaction and SOC, the magnetic moment of the polaron is −0.92 *μ*_*B*_, in which *μ*_*B*_ is the Bohr magneton. The electron in the small molecule keeps nearly total spin polarized with magnetic moment 1.0 *μ*_*B*_ because of the confinement of the size of the small molecule. The spin of the polymer and the small molecule are opposite because of the antiferromagnetic coupling. The net magnetic moment of the system is 0.08 *μ*_*B*_. The spin distribution of the system is shown in Fig. 3a.

The transferred electron may also be distributed among some small molecules. In this case, it is found that the favorable state is that the spins on the molecules arrange parallel. We assume that the electron distributes in *m* adjacent molecules identically, and Fig. 3b shows the relationship between the magnetic moment of the system and *m*. Evidently, with the extension of the transferred electron among the small molecules, the value of the magnetic moment increases.

**Figure 4:**
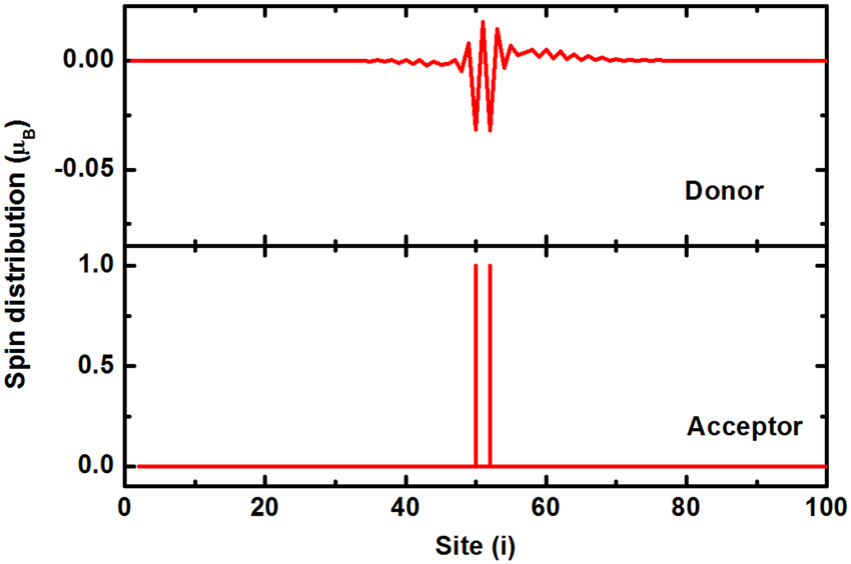
Spin distribution of the system with two electrons transfer.

Then we consider the situation of two electrons transfer from the polymer to the small molecules. Because of the strong e-ph coupling, the left two holes in the polymer will bound together to form a localized bipolaron case. The two transferred electrons tend to close to each other because of the coupling with the localized state in the polymer. Although the bipolaron has a closed-shell structure, there appears a small spin oscillation due to the spin exchange coupling with the small molecules, as showed in Fig. 4. Just because of the spin oscillation, the small molecules take a parallel spin arrangement. The total magnetic moment is about 2.0 *μ*_*B*_ nearly provided by the small molecules, which have open-shell structures in this case.

**Figure 5:**
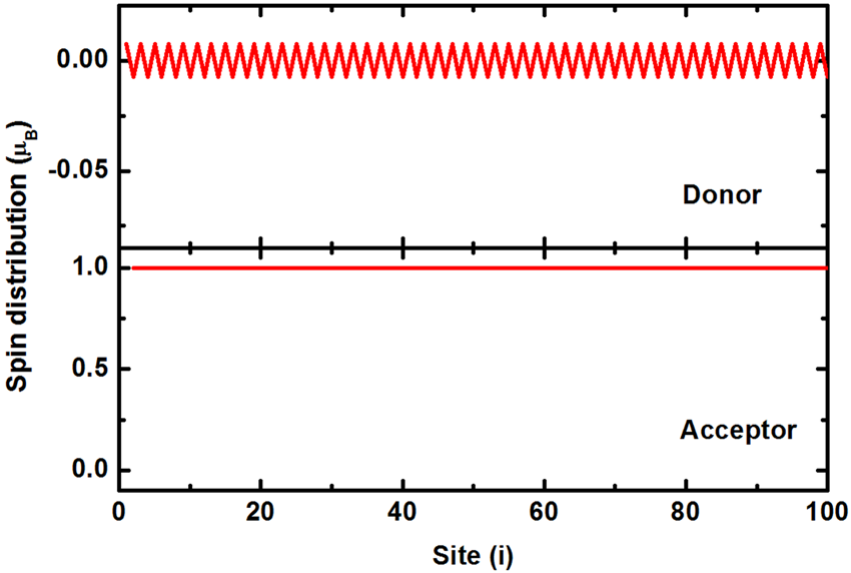
Spin distribution of the ferromagnetic system.

Further, we consider enough charge transfers under photoexcitation. We suppose that each small molecule has one extra electron and its closed-shell structure is broken. The spin polarization distribution of the system is showed in Fig. 5. It is found that the polymer has no net moment but with a weak spin density wave, while the small molecules appear a ferromagnetic order. The total magnetic moment of organic charge transfer composite is basically provided by the small molecules. The ferromagnetic order in the small molecules is stabilized by the spin wave in the polymer through their coupling. The coupling of localized spin magnetic moments through a medium is similar to the Ruderman-Kittel-Kasuya-Yosida (RKKY) interaction in ferromagnetic^35^. Further calculation shows that any damage to the ferromagnetic order will increase the total energy of the whole system.

**Figure 6:**
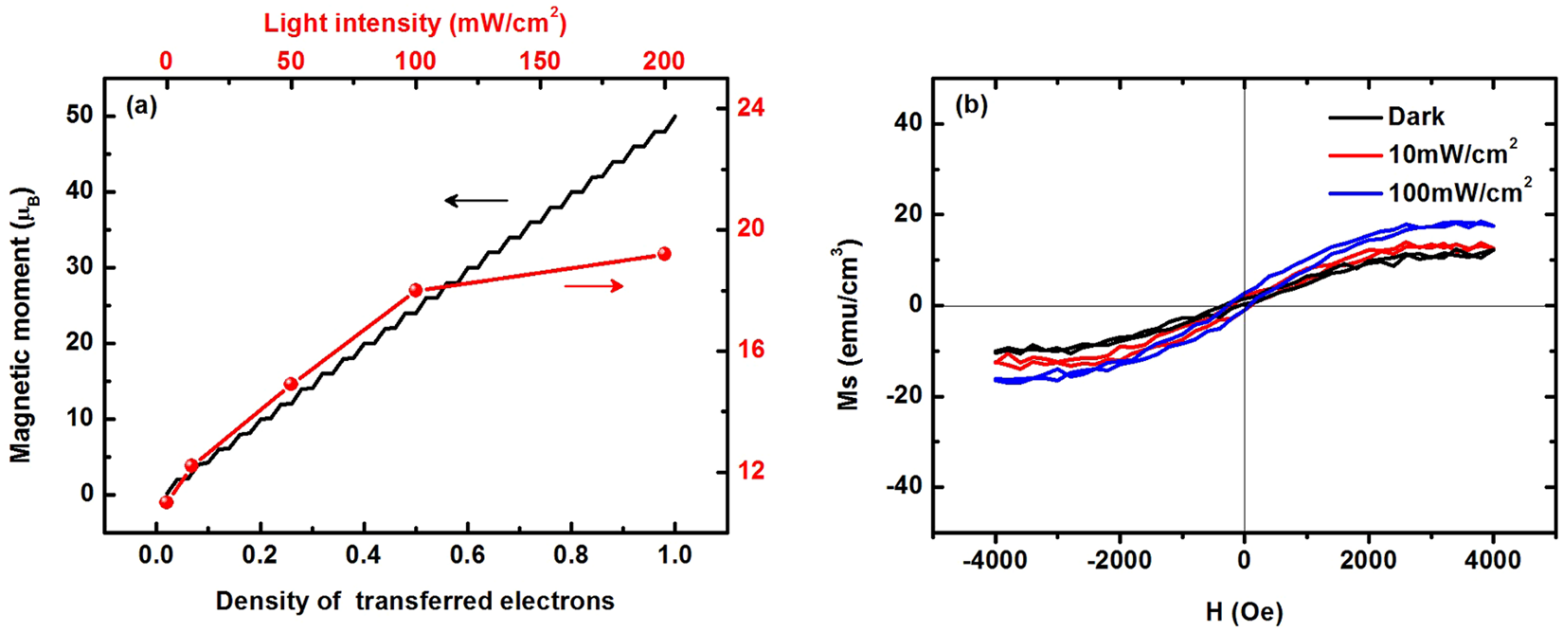
**(a)** Black line: the dependence of the magnetic moment on the density of the transferred electrons (theoretical result). Red line: the dependence of the saturated magnetization on the intensity of the incident light in nw-P3HT/PCBM composite (experimental data). **(b)** The magnetic hysteresis loops with different incident light intensity.

It is known that the number of transferred electrons in organic charge transfer composite is a controllable physical quantity, which could be changed through tuning the interfacial coupling between donor and acceptor^36^ or changing incident light intensity. Theoretically, we study the dependence of the magnetic moment on the density of the transferred electrons. As shown in Fig. 6a (black line), it is found that the magnetic moment increases with the density of the transferred electrons. Experimentally, we also study the dependence of the saturation magnetization *Ms* on the intensity of the incident light in nw-P3HT/PCBM. Figure 6b shows the measured magnetic hysteresis loops composite with different incident light intensity, where we obtain the saturation magnetization at a given incident light. As shown in Fig. 6a (red line), it is found that the magnetization increases with the light intensity. If assuming that the number of transferred electrons is proportional to the light intensity, we find that the theoretical result is well consistent to the experimental data, especially in the regime of low light intensity. The deviation of the experimental data from the theoretical result at high light intensity may be due to that, in the real composite, the excited electrons and holes have a large probability to recombine or annihilate when the density of the transferred charges is high.

## Conclusions

In summary, the spin polarization or ferromagnetism in organic composite nw-P3HT/C_60_ with closed-shell structures is investigated in the frame work of extended Su-Schrieffer-Heeger tight-binding model. We find that there may exist spontaneous charge transfer because of the e-ph coupling in organic materials, which will not realize in inorganic system. To present spin signal, we have to break the closed-shell structure. Electrons can be transferred from polymer to small molecules and transferred electron is spin polarized and its distribution will influence the spin magnetic moment as well as the spin density distribution. The polymer appears a spin oscillation due to the spin exchange coupling with the small molecules even though the polymer contains a spinless structure. Transferred electrons are coupled together through the spin density wave in the polymer and appear a ferromagnetic order. The magnetic moment of the system is mainly provided by the spin polarized small molecules. With the increase of the density of the transferred electrons, the magnetic moment value increases, which is consistent to our experimental observation.

## Methods

### Synthesis of P3HT Nanowire

Firstly, P3HT, purchased from Sigma Aldrich, was dissolved by 1,2-dichlorobenzene (20 mg/mL) in a glovebox (solution preparation is out of air). After P3HT is fully dissolved, 5–10% acetonitrile (Volume ratio) was added into P3HT solution at room temperature, then followed by low power ultrasonic agitation (3–5 min). At last, the solution was placed into the glovebox at room temperature. After 1 day, P3HT nanowire was formed.

### Device Structure

ITO was chosen as the bottom electrode. PEDOT:PSS was coated on it at 3500 rpm for 1 min after the ITO substrate was cleaned. PEDOT:PSS substrates was annealed at 150 °C for 20 minutes in the air. The active layer was the nw-P3HT/PCBM composite with a concentration of 10 mg/mL in 1,2-DCB, which was applied using a spin-coater at 1000 rpm for 1 min. The layer that is about 150 nm thick. Al is chosen as the top electrode through thermal evaporation. The device area is about 2 × 2 mm^2^.

### Magnetic Hysteresis (M-H) Loop Measurements

Vibrating sample magnetometer (VSM) was used to measure M-H loops of the thin film. Laser with 532 nm wavelength is adopted to excited organic devices. The maximum light beam intensity is 200 mW, and beam diameter is 1 mm.
